# Neurological outcome after minimally invasive coronary artery bypass surgery (NOMICS): An observational prospective cohort study

**DOI:** 10.1371/journal.pone.0242519

**Published:** 2020-12-23

**Authors:** Björn Stessel, Kristof Nijs, Caroline Pelckmans, Jeroen Vandenbrande, Jean-Paul Ory, Alaaddin Yilmaz, Pascal Starinieri, Michiel Van Tornout, Nina De Klippel, Paul Dendale

**Affiliations:** 1 Department of Anesthesiology and Pain Medicine, Jessa Hospital, Hasselt, Belgium; 2 Faculty of Medicine and Life Sciences, LCRC, Hasselt University, Diepenbeek, Belgium; 3 Department of Anaesthesiology and Pain Medicine, University Hospital, Leuven, Belgium; 4 Department of Cardiothoracic Surgery, Jessa Hospital, Hasselt, Belgium; 5 Department of Neurology, Jessa Hospital, Hasselt, Belgium; 6 Department of Cardiology, Jessa Hospital, Hasselt, Belgium; IRCCS Policlinico San Donato, ITALY

## Abstract

**Background/Objectives:**

Endoscopic coronary artery bypass grafting (Endo-CABG) is a minimally invasive CABG procedure with retrograde arterial perfusion. The main objective of this study is to assess neurocognitive outcome after Endo-CABG.

**Methods/Design:**

In this prospective observational cohort study, patients were categorised into: Endo-CABG (n = 60), a comparative Percutaneous Coronary Intervention (PCI) group (n = 60) and a healthy volunteer group (n = 60). A clinical neurological examination was performed both pre- and postoperatively, delirium was assessed postoperatively. A battery of 6 neurocognitive tests, Quality of life (QoL) and the level of depressive feelings were measured at baseline and after 3 months. Patient Satisfaction after Endo-CABG was assessed at 3-month follow-up. Primary endpoints were incidence of postoperative cognitive dysfunction (POCD), stroke and delirium after Endo-CABG. Secondary endpoints were QOL, patient satisfaction and the incidence of depressive feelings after Endo-CABG.

**Results:**

In total, 1 patient after Endo-CABG (1.72%) and 1 patient after PCI (1.67%) suffered from stroke during the 3-month follow-up. POCD in a patient is defined as a Reliable Change Index ≤-1.645 or Z-score ≤-1.645 in at least two tests, and was found in respectively 5 and 6 patients 3 months after Endo-CABG and PCI. Total incidence of POCD/stroke was not different (PCI: *n*= 7 [15.9%]; Endo-CABG: *n*= 6 [13.0%], p = 0.732). ICU delirium after Endo-CABG was found in 5 (8.6%) patients. QoL increased significantly three months after Endo-CABG and was comparable with QoL level after PCI and in the control group. Patient satisfaction after Endo-CABG and PCI was comparable. At follow-up, the level of depressive feelings was decreased in all groups.

**Conclusions:**

The incidence of poor neurocognitive outcome, including stroke, POCD and postoperative ICU delirium until three months after Endo-CABG is low and comparable with PCI.

**Trial registration:**

Registered on ClinicalTrials.gov (NCT02979782)

## Background

The most common neurological disorders after cardiac surgery are postoperative cognitive dysfunction (POCD), stroke and delirium [1]. POCD is broadly defined as a decline in cognitive function arising after surgery. Long-term POCD or cognitive decline 3 to 6 months post-surgery occurs in 10-30% of all patients after conventional cardiac surgery and is associated with an increased mortality and major financial consequences [2]. The '1995 statement of consensus on assessment of neurobehavioral outcomes after cardiac surgery' summarizes a list of recommendations to conduct reliable POCD testing, including a battery of the most reliable neuropsychological tests to assess for POCD in clinical studies [3]. Adherence to consensus statement recommendations in studies investigating POCD after cardiac surgery however is very low [4]. The statistical methods used to define the cut-off point between POCD and normal variation in cognitive function also vary widely [5]. The Reliable Change Index (RCI) however is believed to be the most precise method because it relates the changed scores to the normal test-retest variability in an age-matched healthy control population over the same period to correct for practice effects, normal variability and cognitive decline in healthy subjects [5].

Stroke is a severe neurological complication after cardiac surgery with an incidence of 1-6% and is caused by disturbances in the blood supply to the brain [6]. It is diagnosed based on neurological evaluation and signs on CT or MRI scan [7].

Delirium is characterised by acute onset attention disorders and fluctuating changes in the mental state of the patient with an incidence of 5-50% after cardiac surgery [6, 8, 9]. The aetiology of delirium is usually multifactorial. Delirium can easily be diagnosed using the CAM-ICU score (Confusion Assessment Method for the Intensive Care Unit) [10].

New developments in cardiac surgery have led to a shift towards minimally invasive cardiac surgery. The main benefit of minimal invasive surgery may be fast recovery and return to work. Endo-CABG is a new minimally invasive endoscopic coronary artery bypass grafting (CABG) technique to treat patients with single- and multi-vessel coronary artery disease and its technique description and short-term clinical outcomes have recently been published [11]. Endo-CABG avoids median sternotomy by combining a thoracoscopic technique, a mini-thoracotomy and retrograde arterial perfusion (RAP). The RAP however may, at least theoretically, be associated with a higher incidence of neurological complications, including POCD, due to the risk of cerebral embolization, particularly in patients with severe (grades IV and V) aortic atherosclerosis [12].

The main objective of this study is to assess neurocognitive outcome and the incidence of neurological complications after Endo-CABG with RAP.

## Material and methods

### Study design

The complete study protocol has been published in *BMJ open* [13]. Briefly, this observational prospective cohort study was approved by the ethics committee of the Jessa hospital (ethics committee N°: 16.115/anesth16.01, B243201630254, chairperson: Magerman K) on December 1, 2016 and designed to investigate the neurocognitive outcome and the incidence of neurological complications in patients treated with Endo-CABG. An amendment was approved by the ethics committee of the Jessa hospital on December 1, 2017 to increase the sample size from 150 to 180 participants to account for a higher than expected loss-to-follow-up. Informed consent was obtained from all participants. The Conform the “1995 statement of consensus on assessment of neurobehavioral outcomes after cardiac surgery” [3], a comparative and control group were enrolled in a 1:1:1 ratio and age and sex matched to the intervention group (Endo-CABG). Between January 2016 and August 2018, a total of 180 patients were enrolled in this study. The intervention group included consecutive patients treated with elective Endo-CABG following a standardized protocol for anaesthesia, surgical technique and cardiopulmonary bypass to reduce treatment heterogeneity and the risk of poor neurological outcome. Aortic atherosclerosis was assessed with transoesophageal echocardiography (TEE). An optimal blood pressure (with a mean arterial pressure > 70mmHg or even > 80mmHg if significant carotid stenosis was present), central venous pressure < 5 mmHg, normocapnia and normoglycemia were maintained, also during extracorporeal circulation, hemoglobin was kept above 7.5g/dl, and cerebral oxygen saturation was kept close to baseline. The comparative group consisted of patients scheduled for percutaneous coronary intervention (PCI) and was included to correct for normal comorbidities in cardiovascular patients. The decision to treat the patient suffering from coronary artery disease with endo-CABG or PCI was made as routine clinical care by the heart team, which includes the valued opinion of an interventional cardiologist and a cardiovascular surgeon. The heart team selects the best revascularization strategy in accordance with the current guidelines on myocardial revascularization. The control group included healthy age- and sex-matched subjects that were used to correct for the natural variation and the learning effect of repeated neurocognitive testing. Exclusion criteria were a history of postoperative cognitive dysfunction (POCD), delirium or CVA (stroke), symptomatic carotid artery disease, dementia (mini-mental status evaluation [MMSE] <20/30), renal (glomerular filtration rate [GFR] <30 ml/min) or hepatic dysfunction (serum glutamic-oxaloacetic transaminase [SGOT]/ serum glutamic-pyruvic transaminase [SGPT] or aspartate aminotransferase [AST]/alanine aminotransferase [ALT] more than three times upper limit of normal [>3x ULN]), a history of drug and/or alcohol abuse and an inability to perform the neurological test battery due to physical conditions or a language barrier.

#### Outcome measures

The primary objective was to assess the incidence of POCD, stroke, and delirium after Endo-CABG. Secondary outcome measures were quality of life (QoL), patient satisfaction with the procedure, patient satisfaction with the performed neurocognitive tests and the incidence of depressive feelings before Endo-CABG and 3 months after Endo-CABG.

### Test battery

Neurocognitive tests to assess POCD were performed at baseline (2-14 days prior to procedure) and 3 months after surgery/PCI (or baseline testing for control group) by 2 well-trained investigators to guarantee standardized testing (Table 1).

**Table 1:** The neurological test battery.

Test	Domain	Task	Analysis
RAVLT	*Verbal memory*	Immediate recall: 15 words read 5 successive times with each time a free recall.	Total number of words
		Delayed recall: Free recall of the list of 15 words after 30 minutes.	Total number of words
TMT-A	*Attention*	Connect 25 consecutive numbers with a pencil.	Time to complete task
TMT-B	*Attention*, *processing speed*	Connect alternating numbers (1-13) and letters (A-L).	Time to complete task
WAIS-III Digit Span	*Working memory*	Forward: recall of number sequences.	Number of recalled sequences
		Backward: recall of reversed number sequences.	
WAIS-III Symbol Coding	*Processing speed*	Fill out blanks according to a key. Key consists of symbols corresponding to different digits.	Number of filled blanks within 120 seconds
Grooved pegboard	*Motor function*	Insert pegs into grooved pegboard holes. First, perform test with dominant hand. Next, perform test with non-dominant hand.	Time to complete task

RAVLT: Rey Auditory Verbal Learning Test; TMT: Trail-Making Test; WAIS: Wechsler Adult Intelligence Scale.

POCD was defined as a decline in performance on these tests between baseline and 3-month follow-up beyond natural variation and learning effects.

Patients also underwent a neurological examination by a neurologist both pre- and postoperatively to assess the incidence of stroke. In case of clinical suspicion of stroke, brain CT or MRI scan was performed. Patients suffering from stroke within 3 months after surgery/PCI were automatically classified as having POCD.

Delirium was assessed in the intervention group using the Confusion Assessment Method for the Intensive Care Unit (CAM-ICU) tool postoperatively at the ICU daily.

The European Quality of Life-5 Dimensions (EQ-5D) questionnaire and the European Quality of life Visual Analog Scale (EQ VAS) to determine the quality of life, were performed at baseline (2-14 days prior to procedure) and 3 months after surgery/PCI. The Dutch EQ-5D tariff was calculated based on the formula of Mulhern *et al*. [14], using the β-values of Versteegh *et al*. [15].

The Center for Epidemiological Studies Depressions (CES-D) questionnaire was performed to test the mood state of patients.

Patient satisfaction regarding surgery, follow-up and recovery and the performed neurocognitive tests was assessed using an 11-point Numerical Rating Scale (NRS).

In addition to the primary and secondary outcome measures, participants’ age, gender, Body Mass Index (BMI), American Society of Anesthesiologists (ASA) classification, highest level of education and anxiety level measured by the Surgical Fear Questionnaire (SFQ) [16] were also recorded at baseline.

### Calculations & statistical analyses

The primary endpoint, POCD three months after surgery, was defined as ‘a decline in performance on neuropsychological assessment beyond natural variation and learning effects’. The incidence was calculated as a Reliable Change Index (RCI) ≤-1.645 (significance level 5%) or Z-score ≤-1.645 in at least two different tests. The Z-scores and the RCI were used to control for test-retest variability and were calculated as follows: for each patient (x) and control (x_c_), the baseline score from each test was subtracted from the follow-up score, providing Δx and Δx_c_. Afterwards, the Z-scores were calculated by subtracting the mean change of Δx_c_ from Δx and dividing this value by the SD of Δx_c_, to respectively eliminate practice effects and to exclude the effect of natural variation in test performance. Based on the Z-scores, the RCI was calculated by dividing the sum of the Z-scores of the different tests, by the SD of the sum of Z-scores from the control group. Z-scores using time as a test performance score, were inverted to ensure that a negative difference shows a decrease in function. In general, positive values indicate improvement, while negative values indicate decline in overall test performance. Additionally, patients suffering from a stroke within three months after their procedure are were automatically classified as having POCD.

All variables were checked for normal distribution by Shapiro-Wilk test and variables were Log-transformed to satisfy conditions of normality (CES-D, Grooved pegboard dominant & non-dominant, Trail making Test A & Test B). Quality of Life (EQ5D) was analyzed using repeated measures ANOVA, with time and procedure as factors and Fisher’s Least Significant Difference (LSD) post-hoc was applied. Missing values were excluded from the repeated measure ANOVA analyses.

Non-normally distributed data (EQ-5D index, NRS) were analyzed using the non-parametric Kruskal-Wallis and/or Mann Whitney U test.

Data is presented as mean ± SD, unless otherwise specified. Statistical significance was set at *p*<0.05 and all analyses were performed using IBM SPSS v25 (IBM Corporation, Armonk, NY, USA).

The necessary sample size was determined for the primary outcome, i.e. the incidence of POCD after Endo-CABG. An incidence of POCD after CABG and after PCI (comparative group) was assumed in 30% and 7%, respectively [17–20]. Using a two-sided Chi-Square Test with a significance level (alpha) of 0.05, the required sample size was consequently determined to be at least 44 patients per group to obtain a power of 80%. To correct for loss to follow-up (10%), a total of 150 patients were initially required.

Due to an higher than expected rate of loss to follow-up (27%), an amendment to the research protocol was approved by the ethics committee of the Jessa hospital on December 1, 2017 to inflate the study population to a total of 180 patients (60 per group).

## Results

### Patient characteristics

**Fig 1:**
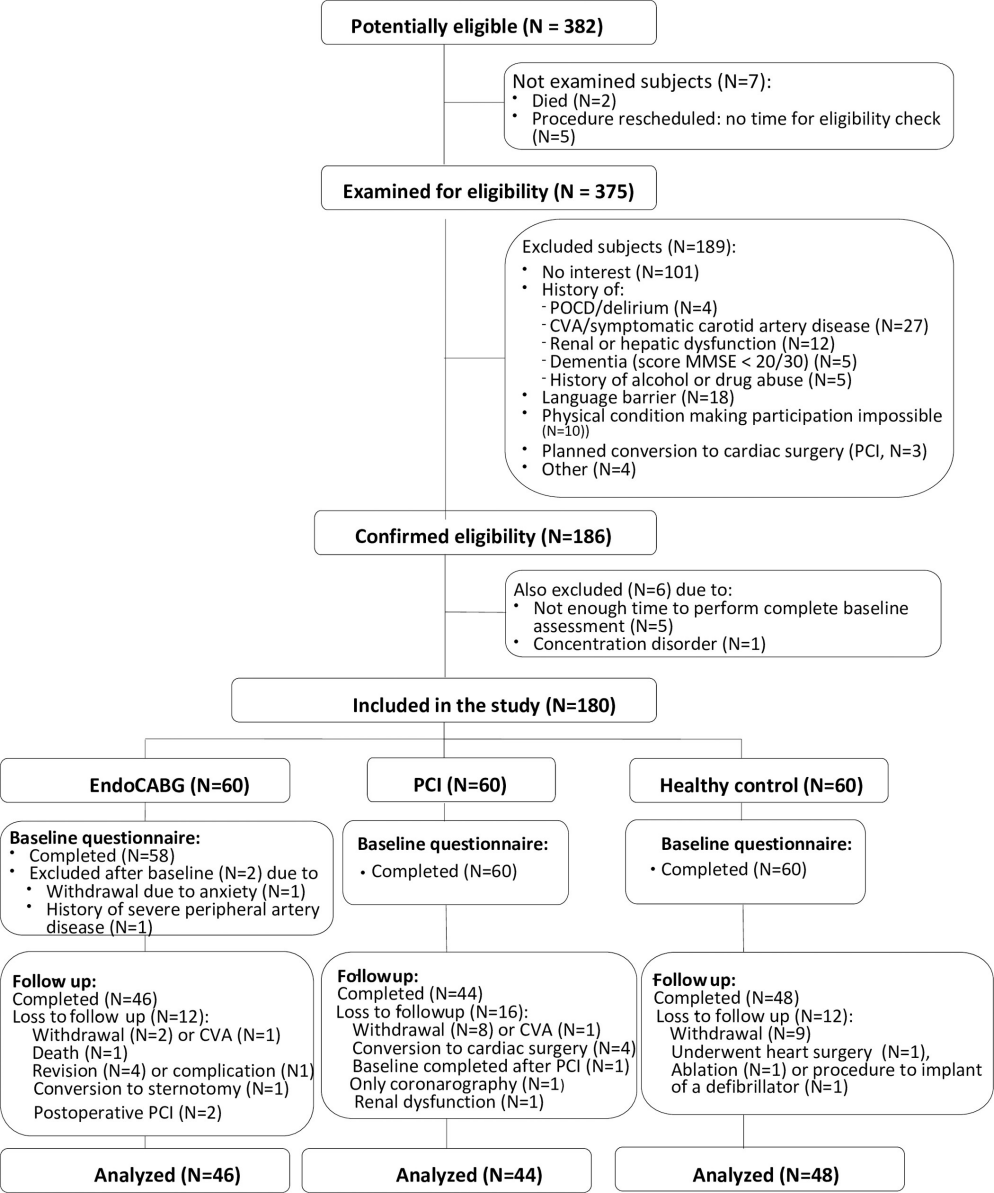
Flow diagram of inclusion and exclusion.

A STROBE flow chart of patient selection and exclusion is presented in Fig 1. One patient had to be converted to sternotomy due to TEE confirmation of severe (grades IV and V) aortic atherosclerosis. Baseline patient characteristics are presented in Table 2 and details related to surgery in Table 3.

**Table 2:** Baseline patient characteristics.

		EndoCABG	PCI	Control
		(N = 46)	(N = 44)	(N = 48)
*Age (years)*		64,61 (9,95)	64,65 (10,27)	64,63 (9,23)
*Male (no*. *(%))*		38 (82,61)	37 (84,09)	38 (79,17)
*BMI (kg*.*m-2)*		27,83 (4,47)	28,44 (5,38)	26,26 (3,52)
*Education (no*. *(%))*				
Primary school		5 (10,87)	1 (2,27)	3 (6,25)
Lower secondary education		8 (17,39)	10 (22,73)	10 (20,83)
Upper secondary education		21 (45,65)	24 (54,55)	16 (33,33)
Higher education		12 (26,09)	9 (20,45)	19 (39,58)
Profession (*no*. *(%))*				
Retired		27 (58,70)	32 (72,73)	30 (62,50)
Working		15 (32,61)	11 (25,00)	15 (31,25)
Unemployed		3 (6,52)	1 (2,27)	0 (0,00)
Unfit to work		1 (2,17)	0 (0,00)	3 (6,25)
*Current smoker (no*. *(%))*		5 (10,87)	12 (27,27)	4 (8,33)
*Alcohol consumer (no*. *(%)*		25 (54,35)	26 (59,09)	33 (68,75)
*Peripheral vascular disease (no*. *(%))*		3 (6,52)	1 (2,27)	-
*Atrial fibrillation (no*. *(%))*		2 (4,34)	6 (13,63)	-
*Diabetes (no*. *(%))*		12 (26,08)	2 (4,54)	-
*Arterial hypertension (no*. *(%))*		25 (54,35)	30 (68.18)	-
*Cholesterol (no*. *(%))*		38 (82,60)	29 (65.91)	-
*COPD (no*. *(%))*		0 (0,00)	3 (6,81)	-
*EUROSCORE*		0,93 (0,58, 1,56)	-	-
*NYHA*		1,00 (1,00, 2,00)	-	-
*EF(%)*		60 (52, 65)	58 (47, 60)	-
*MMSE score (0-30)*		28,43 (1,22)	28,30 (1,67)	28,50 (15,53)
*CES-D (0-60)*		8,22 (7,28)	6,42 (5,02)	5,38 (5,58)
*Fear short-term (0-40)*		13,78 (8,41)	10,88 (7,61)	NA
*Fear long-term (0-40)*		9,72 (7,85)	10,36 (8,46)	NA

**Table 3:** Details related to surgery.

EndoCABG		PCI
(N = 46)		(N = 44)
*Ventilation time (h)*	8,00 (5,00, 11,00)	*-*
*Transfusion (blood)*	0,55 ± 1,55	*-*
*Transfusion (thrombocytes)*	0,23 ± 0,75	*-*
*Transfusion FFP*	0,12 ± 0,45)	*-*
*Blood loss per-op*	274 (239,5, 494,0)	*-*
*Bleeding 24h*	384 (190, 800)	*-*
*ICU LOS (h)*	56 (39, 87)	*-*
*Hospital LOS (days)*	8 (6, 10)	*-*
*Clamp time (min)*	53,50 (40,75, 65,00)	*-*
*Perfusion time (min)*	94,50 (72,25, 106,00)	*-*
*Operation time (min)*	203 (174, 249)	*-*
*Number of stents*		
*1*		24 (54.54)
*2*		13 (29.54)
*3*		5 (11.36)
*Number of bypasses*		
*1*	0 (0,00)	
*2*	24 (52.17)	
*3*	27 (41.30)	
*4*	3 (6.52)	

NYHA: New York Association Functional Classification, COPD: chronic obstructive pulmonary disease, EF: left ventricle ejection fraction, FFP: fresh frozen plasma, LOS: length of stay. Data are presented as median (25^th^, 75^th^ percentile) or as mean ± SD.

#### Stroke after Endo-CABG

In total, 2 patients suffered from a stroke during the 3-month study period, 1 patient after Endo-CABG (1.72%) and 1 patient after PCI (1.67%). The patient in the Endo-CABG group had a history of claudication and symptomatic carotid disease, treated with left carotid endarterectomy. Postoperatively, signs of left hemiplegia and brain CT confirmed the diagnosis of right hemisphere stroke. He recovered completely after a 2-month rehabilitation period.

### POCD after Endo-CABG

POCD, defined as an RCI ≤-1.645 (significance level 5%) or Z-score ≤-1.645 in at least two different tests, was found in respectively 5 and 6 patients 3 months after Endo-CABG and PCI. Hence, combined with the patients diagnosed with stroke, the total incidence of POCD was not different between groups (Endo-CABG: *n*= 6 [13.0%]; PCI: *n*= 7 [15.9%], p = 0.732).

**Fig 2:**
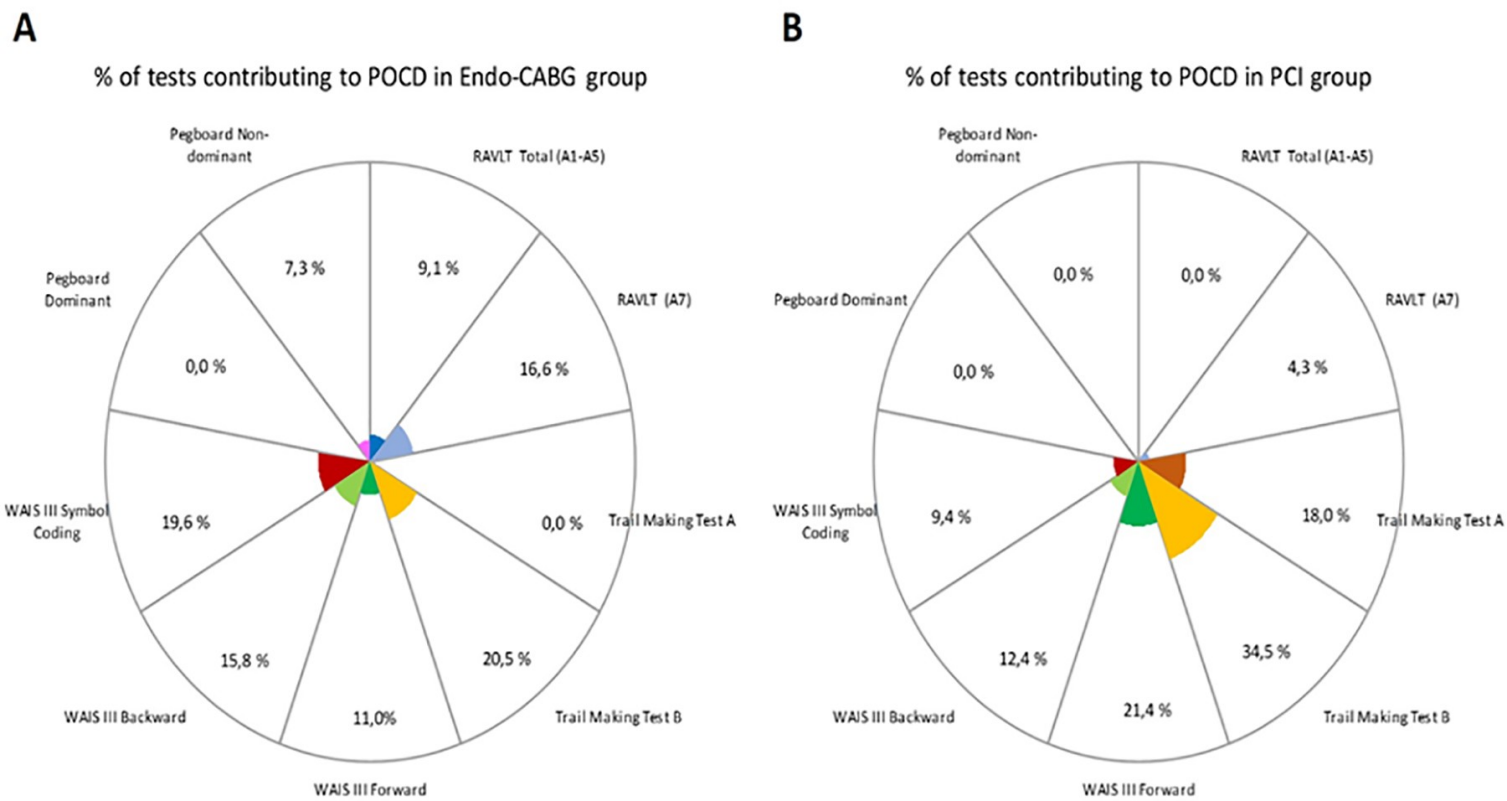
Percentage of tests contributing to POCD. Data are derived from patients with POCD in the Endo-CABG group (*n*=5) (**A**) and the PCI group (*n*=6) (**B**), excluding patients who suffered from a CVA within three months after their procedure (Endo-CABG: *n*=1; PCI: *n*=1).

In the Endo-CABG group, patients with POCD showed the worst decline in their ‘processing speed’ (Trail making test B (20.5%) and WAIS-III Digit symbol coding test (19.6%)) and ‘verbal memory’ (RAVLT delayed recall score (16.6%)) (Fig 2A). Patients with POCD after PCI showed also the worst decline in their ‘processing speed’ (Trail making test B (34.5%)), followed by ‘working memory’ (WAIS-III Digit Span Forward (21.4%) and ‘attention’ (Trail making test A (18.0%)) (Fig 2B).

### Delirium after Endo-CABG

Post-operative delirium at the ICU was found in 5 (8.6%) patients after Endo-CABG.

### Quality of life

Mean EQ-5D VAS score and EQ-5D scores at baseline and at 3-month follow-up are presented in Table 4. At baseline, the EQ VAS score was highest in the control group and significantly different between groups (ptime= <0.001, pgroup = 0.062, ptime*group = 0.036) (Table 5). LSD post-hoc analyses revealed a significant difference in the control group vs. the PCI group (p = 0.002) and vs. the Endo-CABG group (p = 0.033), but not between the PCI and the Endo-CABG group (p = 0.367). After three months, no significant difference was observed between groups.

**Table 4:** Mean EQ-5D VAS score and EQ-5D scores at baseline and at 3-month follow-up.

	CONTROL				Endo-CABG				PCI			
	Baseline		Follow-up		Baseline		Follow-up		Baseline		Follow-up	
	N = 48		N = 48		N = 46		N = 46		N = 44		N = 44	
**EQ-5D VAS score**	**Mean**	**SD**	**Mean**	**SD**	**Mean**	**SD**	**Mean**	**SD**	**Mean**	**SD**	**Mean**	**SD**
	80,0	11,3	81,8	14,2	71,5	14,5	79,5	11,8	74,0	14,3	78,4	13,6
**EQ-5D index score**	0.93	0.1	0.95	0.1	0.89	0.1	0.95	0.1	0.88	0.1	0.92	0.1
**Mobility**	**N**	**(%)**	**N**	**(%)**	**N**	**(%)**	**N**	**(%)**	**N**	**(%)**	**N**	**(%)**
No problems	35,0	72,9	37,0	77,1	33,0	71,7	37,0	80,4	27,0	61,4	29,0	65,9
Slight problem	5,0	10,4	9,0	18,8	6,0	13,0	4,0	8,7	4,0	9,1	7,0	15,9
Moderate problem	4,0	8,3	0,0	0,0	5,0	10,9	4,0	8,7	7,0	15,9	4,0	9,1
Severe Problems	3,0	6,3	1,0	2,1	2,0	4,3	1,0	2,2	6,0	13,6	4,0	9,1
Unable	1,0	2,1	1,0	2,1	0,0	0,0	0,0	0,0	0,0	0,0	0,0	0,0
**Self-Care**												
No problems	47,0	97,9	48,0	100,0	44,0	95,7	44,0	95,7	40,0	90,9	40,0	90,9
Slight problem	1,0	2,1	0,0	0,0	0,0	0,0	2,0	4,3	1,0	2,3	3,0	6,8
Moderate problem	0,0	0,0	0,0	0,0	2,0	4,3	0,0	0,0	3,0	6,8	1,0	2,3
Severe Problems	0,0	0,0	0,0	0,0	0,0	0,0	0,0	0,0	0,0	0,0	0,0	0,0
Unable	0,0	0,0	0,0	0,0	0,0	0,0	0,0	0,0	0,0	0,0	0,0	0,0
**Usual Activities**												
No problems	46,0	95,8	47,0	97,9	38,0	82,6	42,0	91,3	32,0	72,7	34,0	77,3
Slight problem	2,0	4,2	1,0	2,1	5,0	10,9	3,0	6,5	5,0	11,4	9,0	20,5
Moderate problem	0,0	0,0	0,0	0,0	3,0	6,5	1,0	2,2	3,0	6,8	1,0	2,3
Severe Problems	0,0	0,0	0,0	0,0	0,0	0,0	0,0	0,0	4,0	9,1	0,0	0,0
Unable	0,0	0,0	0,0	0,0	0,0	0,0	0,0	0,0	0,0	0,0	0,0	0,0
**Pain/Discomfort**												
No pain	38,0	79,2	33,0	68,8	27,0	58,7	23,0	50,0	32,0	72,7	29,0	65,9
Slight pain	5,0	10,4	9,0	18,8	14,0	30,4	16,0	34,8	7,0	15,9	8,0	18,2
Moderate pain	3,0	6,3	5,0	10,4	4,0	8,7	3,0	6,5	4,0	9,1	5,0	11,4
Severe pain	1,0	2,1	1,0	2,1	1,0	2,2	4,0	8,7	1,0	2,3	2,0	4,5
Extreme pain	1,0	2,1	0,0	0,0	0,0	0,0	0,0	0,0	0,0	0,0	0,0	0,0
**Anxiety/Depression**												
not anxious	39,0	81,3	45,0	93,8	26,0	56,5	45,0	97,8	32,0	72,7	41,0	93,2
slightly anxious	9,0	18,8	3,0	6,3	16,0	34,8	1,0	2,2	9,0	20,5	1,0	2,3
moderately anxious	0,0	0,0	0,0	0,0	2,0	4,3	0,0	0,0	3,0	6,8	2,0	4,5
severely anxious	0,0	0,0	0,0	0,0	2,0	4,3	0,0	0,0	0,0	0,0	0,0	0,0
extremely anxious	0,0	0,0	0,0	0,0	0,0	0,0	0,0	0,0	0,0	0,0	0,0	0,0

SD: standard deviation; EQ-5D: European Quality of Life-5 Dimensions. The EQ-5D VAS score is a vertical visual analogue scale and assesses the respondent´s current state of health between 100 (best imaginable health) and 0 (worst imaginable health).

**Table 5:** Repeated measures ANOVA mean EQ5D VAS scores.

	Mean Square	F-statistic	p-value
Time	1565.887	23.003	<0.001
Time*Group	232.174	3.411	0.036
Group	817.413	2.838	0.062

EQ-5D: European Quality of Life-5 Dimensions. VAS: Visual Analogue Scale. A p-value <0.05 is considered statistically significant.

### Patient´s mood state

At baseline, the level of depressive feelings measured by the CES-D (score 0 – 60 with 0 = no depression at all and cut-off point for depression = 16) was not significantly different between groups (Control: 5.38 ± 5.6; PCI: 6.42 ± 5.0; Endo-CABG: 8.22 ± 7.3; p = 0.07). At follow-up, the level of depressive feelings was decreased in all groups with no differences between groups (Control: 4.60 ± 5.3; PCI: 4.93 ± 5.0; Endo-CABG: 4.76 ± 4.7; p_time_= 0.001, p_group_=0.789, p_time*group_=0.642).

### Patient satisfaction

There were no differences between the PCI and the Endo-CABG group with satisfaction regarding ‘surgery and care’ (PCI: 8.53 ± 1.3; Endo-CABG: 8.34 ± 1.5; p = 0.666), ‘post-operative follow-up and recovery’ (PCI: 8.19 ± 1.4; Endo-CABG: 8.16 ± 1.8; p = 0.699) and ‘neurocognitive testing’ (PCI: 8.74 ± 1.1; Endo-CABG: 8.98 ± 0.8; p = 0.464).

## Discussion

Our results suggest that the risk of stroke after Endo-CABG (1.72%) is comparable with the risk of stroke after PCI (1.67%). Furthermore, the incidence of POCD, three months after Endo-CABG was rather low (13.0%) and not statistically different from the incidence of POCD three months after PCI (15.9%,p = 0.732). The incidence of postoperative delirium at the ICU was also low (8.6%). Patient satisfaction was comparable between the Endo-CABG group and the PCI group and the level of depressive feelings decreased in both groups, three months after the procedure.

The incidence of POCD 3 to 6 months after on-pump cardiac surgery varies widely in literature. For example, 3 months after elective conventional CABG, Toeg et al. found an incidence of POCD of 19% (132/696) [18]. Kok et al. demonstrated an incidence of POCD of 26% (15/57) 3 months after elective CABG [19]. Hernandez et al. even documented an incidence of POCD of 47.1% (48/102), 6 months after on-pump CABG [20]. Thus, compared to literature, the present study found a low incidence of POCD (13% - 6/46), 3 months after elective Endo-CABG. These differences could be attributed to variations in research methods, i.e. applied test battery and statistical methods [4, 5]. It should be noted that in the current study POCD assessment was in accordance with Consensus Statement recommendations and that state-of-the-art analytic criteria for POCD have been applied [4, 5]. Moreover, the incidence of POCD was not different between the Endo-CABG and the PCI group. This might not be surprising as there is increasing evidence that underlying patient factors such as ischemic cardiac disease and pre-procedural inflammatory state are risk factors for cognitive decline and stroke regardless of revascularisation method [21]. Furthermore, a recent retrospective analysis demonstrated that 30.8% of PCI patients suffered persistent high inflammatory states for more than four weeks after stent implantation. All-cause mortality in these patients was 3.8 times higher than in low inflammatory state PCI patients [22]. Compared to the incidence of 5-50% of delirium after cardiac surgery described in literature [6, 8, 9], the incidence of delirium after Endo-CABG (8.6%) was also rather low. This might be explained by strong adherence to the delirium prevention protocol at our ICU which started in 2014 as a quality improvement measure. Finally, only 1 (1.72% - 1/58) patient suffered a stroke in the Endo-CABG group which is well in range of the reported stroke incidence of 0.7-2.9% after conventional CABG [20, 23]. Moreover, also one patient in the PCI-group suffered from stroke. These observations may prove that the Endo-CABG procedure using RAP in patients without severe aortic sclerosis on TEE does not increase the risk of neurological complications despite the use of RAP.

Endeavors were made to optimize the anaesthetic protocol for Endo-CABG at our center. This protocol focusses on maintaining an optimal, personalized blood pressure and maintaining central venous pressure < 5 mmHg during surgery, keeping hemoglobin above 7.5g/dl, aiming for normocapnia and normoglycemia and keeping cerebral oxygen saturation close to baseline. Recent literature confirms the benefit of these interventions on neurological outcome after cardiac surgery [6]. Also at the ICU, neurologic protection measures were taken, consisting of early mobilization and avoiding hyperthermia. In literature, there is no consensus on optimal, personalized blood pressure level during cardiac surgery to avoid poor neurologic outcome [24, 25]. However, Sun et al. proved that stroke is strongly associated with sustained mean arterial pressure (MAP) of less than 64 mmHg during cardiopulmonary bypass [25]. Therefore, we advocate for maintaining a MAP > 70 mmHg during cardiopulmonary bypass.

It has been suggested that poor neurological outcome may be associated with longer duration of cardiopulmonary bypass [26]. Due to surgical swiftness, mean occlusion time during Endo-CABG is only approximately 50 minutes at our center, which may partly explain the positive results of this study. However, the significance of perfusion time on neurological outcome has also been questioned [27]. In line with this opinion, Lamy et al. couldn’t detect a significant difference in stroke incidence 30 days after off-pump or on-pump CABG [28].

QoL is currently considered a marker of therapeutic effectiveness. This is the first study to date that assessed QoL after Endo-CABG. Compared with baseline, QoL was significantly higher three months after Endo-CABG and the level of QoL three months after Endo-CABG was comparable with the level of QoL three months after PCI and the level of QoL in the control group. Our findings are in line with literature. Bonaros et al. assessed QoL 3 months after totally endoscopic robotic CABG and demonstrated significantly better QoL scores related to bodily pain and physical health compared to conventional CABG [29]. It has also been shown that QoL improves after CABG, even in elderly patients [30]. In contrast, Eefting et al. found significantly higher QoL 1 month after PCI compared to off-pump CABG but this difference was no longer present at 1 year [31].

Our study contains some limitations. First, ideally, the neurocognitive outcome of patients undergoing Endo-CABG should be compared to patients undergoing conventional CABG in a randomised controlled trial. However, conventional CABG surgery is only performed in acute setting and in patients with atherosclerotic disease grade IV or V. Second, this study has only been powered to assess the incidence of POCD after cardiac surgery. Due to the low incidence of stroke after CABG, it is almost not possible to power a study for this primary endpoint. Third, due to an higher than expected rate of loss to follow-up in this study, we were forced to inflate the study population to a total of 180 patients during the course of the study.

Finally, Endo-CABG was performed by only one surgeon in this trial. Therefore, the generalizability of our results can be questioned.

In conclusion, the present study suggests that the incidence of poor neurocognitive outcome, including stroke, POCD and postoperative ICU delirium, until three months after Endo-CABG is low. Moreover, the incidence of stroke and POCD 3 months after Endo-CABG and PCI are comparable. Consequently, theoretical concerns regarding the risk of cerebral embolic complications during RAP in patients with mild atherosclerotic disease grade I,II a III are not justified.

## Supporting information

S1 File Published study protocol. (PDF)

S2 File Original study protocol. (DOCX)

S3 File STROBE checklist for cohort studies. (DOC)

## Abbreviations

POCD: postoperative cognitive dysfunction
RCI: Reliable Change Index
CAM-ICU: Confusion Assessment Method for the Intensive Care Unit
CABG: coronary artery bypass grafting
RAP: retrograde arterial perfusion
PCI: percutaneous coronary intervention
QoL: quality of life
RAVLT: Rey Auditory Verbal Learning Test
TMT: Trail-Making Test
WAIS: Wechsler Adult Intelligence Scale
EQ-5D: European Quality of Life-5 Dimensions
VAS: Visual Analog Scale
CES-D: Center for Epidemiological Studies Depressions
NRS: Numerical Rating Scale
BMI: body mass index
ASA classification: American Society of Anesthesiologists classification
SFQ: Surgical Fear Questionnaire
LSD: Fisher’s Least Significant Difference
MMSE: mini-mental status evaluation

## References

[1] Stamou SC. Stroke and encephalopathy after cardiac surgery: the search for the holy grail. Stroke. Februari 2006;37(2):284–5. 10.1161/01.STR.0000199048.87363.aa 16373633

[2] Newman MF, Grocott HP, Mathew JP, White WD, Landolfo K, Reves JG, et al Report of the substudy assessing the impact of neurocognitive function on quality of life 5 years after cardiac surgery. Stroke. 1 12 2001;32(12):2874–81. 10.1161/hs1201.099803 11739990

[3] Murkin JM, Newman SP, Stump DA, Blumenthal JA. Statement of consensus on assessment of neurobehavioral outcomes after cardiac surgery. Ann Thorac Surg. mei 1995;59(5):1289–95. 10.1016/0003-4975(95)00106-u 7733754

[4] Rudolph JL, Schreiber KA, Culley DJ, McGlinchey RE, Crosby G, Levitsky S, et al Measurement of post-operative cognitive dysfunction after cardiac surgery: a systematic review. Acta Anaesthesiol Scand. Juli 2010;54(6):663–77. 10.1111/j.1399-6576.2010.02236.x 20397979 PMC2919360

[5] van Harten AE, Scheeren TWL, Absalom AR. A review of postoperative cognitive dysfunction and neuroinflammation associated with cardiac surgery and anaesthesia. Anaesthesia. maart 2012;67(3):280–93. 10.1111/j.1365-2044.2011.07008.x 22321085

[6] Liu Y, Chen K, Mei W. Neurological complications after cardiac surgery: anesthetic considerations based on outcome evidence. Curr Opin Anaesthesiol. Oktober 2019;32(5):563–7. 10.1097/ACO.0000000000000755 31145196 PMC6735528

[7] Chalela JA, Kidwell CS, Nentwich LM, Luby M, Butman JA, Demchuk AM, et al Magnetic resonance imaging and computed tomography in emergency assessment of patients with suspected acute stroke: a prospective comparison. Lancet. 27 Januari 2007;369(9558):293–8. 10.1016/S0140-6736(07)60151-2 17258669 PMC1859855

[8] Deiner S, Silverstein JH. Postoperative delirium and cognitive dysfunction. Br J Anaesth. 12 2009;103 Suppl 1:i41–46. 10.1093/bja/aep291 20007989 PMC2791855

[9] Saczynski JS, Marcantonio ER, Quach L, Fong TG, Gross A, Inouye SK, et al Cognitive trajectories after postoperative delirium. N Engl J Med. 5 Juli 2012;367(1):30–9. 10.1056/NEJMoa1112923 22762316 PMC3433229

[10] Gusmao-Flores D, Salluh JIF, Chalhub RÁ, Quarantini LC. The confusion assessment method for the intensive care unit (CAM-ICU) and intensive care delirium screening checklist (ICDSC) for the diagnosis of delirium: a systematic review and meta-analysis of clinical studies. Crit Care. 3 Juli 2012;16(4):R115 10.1186/cc11407 22759376 PMC3580690

[11] Yilmaz A, Robic B, Starinieri P, Polus F, Stinkens R, Stessel B. A new viewpoint on endoscopic CABG: technique description and clinical experience. J Cardiol. 8 Januari 2020; 10.1016/j.jjcc.2019.11.007 31926795

[12] Modi P, Chitwood WR. Retrograde femoral arterial perfusion and stroke risk during minimally invasive mitral valve surgery: is there cause for concern? Ann Cardiothorac Surg. 11 2013;2(6):E1 10.3978/j.issn.2225-319X.2013.11.13 24350000 PMC3857048

[13] Nijs K, Vandenbrande J, Vaqueriza F, Ory J-P, Yilmaz A, Starinieri P, et al Neurological outcome after minimal invasive coronary artery surgery (NOMICS): protocol for an observational prospective cohort study. BMJ Open. 6 Oktober 2017;7(10):e017823 10.1136/bmjopen-2017-017823 28988183 PMC5640084

[14] Mulhern B, Feng Y, Shah K, Janssen MF, Herdman M, van Hout B, et al Comparing the UK EQ-5D-3L and English EQ-5D-5L Value Sets. Pharmacoeconomics. 2018;36(6):699–713. 10.1007/s40273-018-0628-3 29476363 PMC5954043

[15] M Versteegh M, M Vermeulen K, M A A Evers S, de Wit GA, Prenger R, A Stolk E. Dutch Tariff for the Five-Level Version of EQ-5D. Value Health. 2016;19(4):343–52. 10.1016/j.jval.2016.01.003 27325326

[16] Theunissen M, Peters ML, Schouten EGW, Fiddelers AAA, Willemsen MGA, Pinto PR, et al Validation of the surgical fear questionnaire in adult patients waiting for elective surgery. PLoS ONE. 2014;9(6):e100225 10.1371/journal.pone.0100225 24960025 PMC4069058

[17] Sweet JJ, Finnin E, Wolfe PL, Beaumont JL, Hahn E, Marymont J, et al Absence of cognitive decline one year after coronary bypass surgery: comparison to nonsurgical and healthy controls. The Annals of thoracic surgery. 2008;85(5):1571–8. 10.1016/j.athoracsur.2008.01.090 18442540

[18] Toeg HD, Nathan H, Rubens F, Wozny D, Boodhwani M. Clinical impact of neurocognitive deficits after cardiac surgery. J Thorac Cardiovasc Surg. Juni 2013;145(6):1545–9. 10.1016/j.jtcvs.2013.02.061 23535152

[19] Kok WF, Koerts J, Tucha O, Scheeren TWL, Absalom AR. Neuronal damage biomarkers in the identification of patients at risk of long-term postoperative cognitive dysfunction after cardiac surgery. Anaesthesia. Maart 2017;72(3):359–69. 10.1111/anae.13712 27987229

[20] Hernandez F, Brown JR, Likosky DS, Clough RA, Hess AL, Roth RM, et al Neurocognitive outcomes of off-pump versus on-pump coronary artery bypass: a prospective randomized controlled trial. Ann Thorac Surg. 12 2007;84(6):1897–903. 10.1016/j.athoracsur.2007.07.036 18036904

[21] Murkin JM. Panvascular inflammation and mechanisms of injury in perioperative CNS outcomes. Semin Cardiothorac Vasc Anesth. 9 2010;14(3):190–5. 10.1177/1089253210378177 20656746

[22] Kalkman DN, Aquino M, Claessen BE, Baber U, Guedeney P, Sorrentino S, et al Residual inflammatory risk and the impact on clinical outcomes in patients after percutaneous coronary interventions. Eur Heart J. 07 2018;39(46):4101–8. 10.1093/eurheartj/ehy633 30358832

[23] Shroyer AL, Grover FL, Hattler B, Collins JF, McDonald GO, Kozora E, et al On-pump versus off-pump coronary-artery bypass surgery. N Engl J Med. 5 11 2009;361(19):1827–37. 10.1056/NEJMoa0902905 19890125

[24] Vedel AG, Holmgaard F, Rasmussen LS, Langkilde A, Paulson OB, Lange T, et al High-Target Versus Low-Target Blood Pressure Management During Cardiopulmonary Bypass to Prevent Cerebral Injury in Cardiac Surgery Patients: A Randomized Controlled Trial. Circulation. 24 2018;137(17):1770–80. 10.1161/CIRCULATIONAHA.117.030308 29339351

[25] Sun LY, Chung AM, Farkouh ME, van Diepen S, Weinberger J, Bourke M, et al Defining an Intraoperative Hypotension Threshold in Association with Stroke in Cardiac Surgery. Anesthesiology. 2018;129(3):440–7. 10.1097/ALN.0000000000002298 29889106

[26] Baker RA, Hallsworth LJ, Knight JL. Stroke after coronary artery bypass grafting. Ann Thorac Surg. november 2005;80(5):1746–50. 10.1016/j.athoracsur.2005.04.059 16242450

[27] Floyd TF, Shah PN, Price CC, Harris F, Ratcliffe SJ, Acker MA, et al Clinically silent cerebral ischemic events after cardiac surgery: their incidence, regional vascular occurrence, and procedural dependence. Ann Thorac Surg. Juni 2006;81(6):2160–6. 10.1016/j.athoracsur.2006.01.080 16731147

[28] Lamy A, Devereaux PJ, Prabhakaran D, Taggart DP, Hu S, Paolasso E, et al Off-pump or on-pump coronary-artery bypass grafting at 30 days. N Engl J Med. 19 4 2012;366(16):1489–97. 10.1056/NEJMoa1200388 22449296

[29] Bonaros N, Schachner T, Wiedemann D, Oehlinger A, Ruetzler E, Feuchtner G, et al Quality of life improvement after robotically assisted coronary artery bypass grafting. Cardiology. 2009;114(1):59–66. 10.1159/000212115 19365117

[30] Peric V, Jovanovic-Markovic S, Peric D, Rasic D, Novakovic T, Dejanovic B, et al Quality of Life in Patients of Different Age Groups before and after Coronary Artery By-Pass Surgery. Ann Thorac Cardiovasc Surg. 2015;21(5):474–80. 10.5761/atcs.oa.15-00041 26328597 PMC4904857

[31] Eefting F, Nathoe H, van Dijk D, Jansen E, Lahpor J, Stella P, et al Randomized comparison between stenting and off-pump bypass surgery in patients referred for angioplasty. Circulation. 9 12 2003;108(23):2870–6. 10.1161/01.CIR.0000100723.50363.2C 14656913

