# Supplementary material for: Neurological outcome after minimally invasive coronary artery bypass surgery (NOMICS): An observational prospective cohort study
Source: PLoS One. 2020 Dec 23;15(12):e0242519. doi: 10.1371/journal.pone.0242519 (PMC7757846; doi:10.1371/journal.pone.0242519)
Supplement: S2 File — (DOCX) [file pone.0242519.s002.docx]

Neurological outcome after minimal invasive cardiac surgery: a single-centre prospective cohort study

# Onderzoeker(s)

De dagelijkse activiteiten van de studie zullen uitgevoerd worden door Fidel Vaqueriza, student 2e master klinisch moleculaire wetenschappen aan de Universiteit Hasselt en Caroline Pelckmans,. De begeleidende onderzoekers zijn Dr. Björn Stessel en Ingrid Arijs, zij zullen Fidel Vaqueriza en Caroline Pelckmans begeleiden op dagelijkse basis en controleren of de studie op de juiste manier uitgevoerd zal worden.

# Departement

Het uitvoerende departement is de dienst Anesthesie en intensive care in het Jessa ziekenhuis in Hasselt. De academische instelling die deze studie ondersteunt is de Universiteit Hasselt in Diepenbeek.

**Inhoud**

1. Introductie
2. Doel van de studie
3. Eindpunten
   1. Primaire eindpunten
   2. Secundaire eindpunten
4. Studieopzet
5. Studiepopulatie
   1. Populatie
   2. Inclusiecriteria
   3. Exclusiecriteria
   4. Demografische gegevens
6. Studieprocedure
   1. Pre-operatief
   2. Post-operatief
7. Studie analyse
   1. Studie analyse
8. Peri-operatieve procedure
9. Ethische overwegingen en wettelijk kader
10. Bijlagen
11. Referenties

# Introductie

De laatste decennia is er een grote vooruitgang geboekt op het vlak van cardiochirurgische technieken, perfusiesystemen en perioperatief beleid tijdens cardiochirurgie. Dit heeft geleid tot kortere ziekenhuisopnames, verlaagde mortaliteit en een verbetering van de levenskwaliteit van de patiënt na cardiochirurgie. (1;2). Daarentegen blijven neurologische condities als cerebrovasculair accident (CVA of beroerte), delirium en postoperatieve cognitieve dysfunctie (POCD) ) relatief frequent voorkomen na cardiale chirurgie (3;4). Deze neurologische aandoeningen hebben een negatief effect op de kwaliteit van leven, leiden tot extra kosten voor de gezondheidszorg, een verlengde hospitalisatie, alsook een verhoogde mortaliteit na cardiale chirurgie. (5;6;7).

Delirium na cardiale chirurgie komt voor in 5 tot 15% van de patiënten (25). Delirium wordt gedefinieerd als een fluctuerende verandering in mentale status met een acute onset en gekarakteriseerd door een verlaagd bewustzijn van de omgeving en door aandachtstoornissen (10). Postoperatieve symptomen van delirium verschijnen meestal tussen de 1 en 3 dagen na de operatie. In 90% van de gevallen van delirium, gaat het om een transiënte vorm. Maar in 10% van de gevallen leidt delirium tot een verhoogde afhankelijkheid, een verhoogde mortaliteit en een meer persistente cognitieve dysfunctie. Delirium wordt gediagnostiseerd aan de hand van de CAM-ICU (Confusion Assessment Method for the Intensive Care Unit) test. Deze test geeft aan of de patiënt een verminderde aandacht heeft en scoort de patient ofwel positief voor delirium ofwel negatief. Deze test wordt standaard uitgevoerd na chirurgie.

Cerebrovasculair accident (CVA of beroerte) wordt veroorzaakt door een verstoring van de bloedvoorziening aan de hersenen. In ongeveer 80% gaat het om een herseninfarct of ischemisch CVA, veroorzaakt door een verstopt bloedvat (omwille van een trombus of embolie) of hypoperfusie van de hersenen. In 20% heeft men te maken met een hersenbloeding of hemorragisch CVA ten gevolge van een gescheurd bloedvat in de hersenen. Dit zorgt voor hersenschade omwille van verlaagde zuurstof en glucose voorziening aan de hersenen. Bij cardiale chirurgie kan een ischemisch CVA voorkomen omwille van embolisatie van plaques tijdens de ingreep. De incidentie na cardiale chirurgie ligt tussen de 0.8-5.2%, afhankelijk van type ingreep. Het risico op CVA is het hoogst tijdens de eerste dagen na cardiale chirurgie en is makkelijk te diagnosticeren (3). CVA wordt gedefinieerd als plotse dood van bepaalde hersencellen door een tekort aan zuurstof, veroorzaakt door een blokkage in de hersenbloedtoevoer of een ruptuur van een hersenarterie. Bij vermoeden van CVA wordt er een CT of MRI scan afgenomen van de hersenen om CVA te diagnosticeren.

Postoperatieve cognitieve dysfunctie (POCD) is een van de meest voorkomende complicaties na cardiale chirurgie en wordt vaak omschreven als een verstoring van de hersenfunctie causaal gerelateerd aan chirurgie (8;9;10). De oorzaak van deze aandoening is waarschijnlijk multifactorieel: milde cerebrale ischemie omwille van microembolisatie en/of hypoperfusie, een systemische inflammatoire respons uitgelokt door majeure chirurgie, leeftijd en predisponerende neurologische aandoeningen (11;12;13).

Verschillende hersenfuncties worden aangetast bij POCD. De belangrijkste domeinen die aangetast worden zijn: aandacht, cognitieve psychomotor snelheid, taal, leren en geheugen, en executieve functies (9). POCD na cardiale chirurgie heeft een incidentie tussen de 10-50%, afhankelijk van de testgroep en de testomstandigheden (bv. Type operatie, operatieduur) (14;15).

Aangezien POCD vaag gedefinieerd is, multifactorieel is en er nog geen standaardprocedure bestaat om POCD te definiëren, is het zeer moeilijk om juist te achterhalen wat de exacte incidentie is. (16). Er worden verschillende analytische criteria gebruikt om POCD van gewone variaties in cognitieve functie te onderscheiden. De historisch meest gebruikte criteria waren enerzijds een verschil in percentage ten opzichte van de baseline score (meestal >20% in 2 of meer testen) en anderzijds een absolute verlaging ten opzichte van de baseline score (groter dan een vooraf gedefinieerde proportie van de standard afwijking (SD) van 2 of meer testen; vaak >1 SD) (26). Meer recent wordt er gebruik gemaakt van de zogenaamde Reliable Change Index (RCI). Deze index wordt beschreven als een Z-score. POCD wordt hier gedefinieerd als een Z-score lager dan -1.96 op 2 of meer testen.

Om meer homogeniteit te creëren binnen het onderzoek naar POCD na cardiale heelkunde, werd in 1995 de ‘1995 statement of consensus on assessment of neurobehavioral outcomes after cardiac surgery’ voorgesteld (20). Hierin wordt een batterij aan testen voorgesteld die op de meest betrouwbare manier POCD kunnen diagnosticeren. Deze batterij bestaat uit 4 testen: de rey auditory verbal learning test voor verbaal geheugen, trailmaking A en B voor aandacht en de grooved pegboard test voor psychomotor functie (20;21;22). Verder worden ook de Digit span test en de Digit symbol-coding test geïncorporeerd. De Digit span test wordt gebruikt om het korte termijn verbale geheugen te testen, de Digit symbol-coding test wordt gebruikt om verwerkingssnelheid en geheugen te testen. Andere aanbevelingen van deze statement zijn: afnemen van baseline testen en op zijn minst 1 assessment 3 maanden na cardiale heelkunde (waarbij rekening wordt gehouden met een eventueel “learning effect”, incorporatie van een controlegroep in het study design, assessment van angst en depressie (aangezien deze een invloed kunnen hebben op het resultaat van neurofysiologische testen), afnemen van de testen door 1 enkele persoon en afnemen van een neurologisch klinisch onderzoek zowel voor als na de operatie (20).

Zowel CVA, delier als POCD hebben rechtstreekse gevolgen voor de patiënt. De Patiënt zijn cognitieve functies kunnen voor langdurige periode aangetast worden. Dit heeft als gevolg dat de levenskwaliteit van de patiënt vaak achteruit gaat en dat de Patiënt vaak op sociaal vlak ook schade ondervindt. Dit leidt tot teruggetrokkenheid uit de omgeving, verlies van job of een vervroegd verlaten van de arbeidsmarkt. Ook verlengde hospitalisatie periodes en een verhoogde mortaliteit worden waargenomen ten gevolge van neurologische dysfuncties na cardiale chirurgie (6;17;18)

De incidentie van POCD, delier en CVA na conventionele cardiale chirurgie via een klassieke sternotomie is reeds uitvoerig bestudeerd (14;15). De incidentie van POCD, delier en CVA na minimaal invasieve cardiale chirurgie (MICC) is echter nog niet goed bestudeerd. Op theoretische gronden kan men wel aannemen dat het risico op POCD, delier en CVA na MICC negatief wordt beïnvloed door het feit dat men tijdens MICC standaard gebruik maakt van retrograde aorta perfusie. Uiteraard zal een ernstig verkalkte aorta (atheroscerose graad IV en V) een hoger risico met zich meebrengen op embolisatie van plaques naar de cerebrale circulatie (19).

In het JESSA ziekenhuis wordt sinds kort gebruik gemaakt van een revolutionair minimaal invasief extra-corporeel systeem (Minimal invasive Extra-Corporeal Circulation of MiECC). De invloed van dit systeem op de neurologische outcome is ook nog niet duidelijk.

Het primaire doel van deze studie is het bestuderen van de incidentie van delier, POCD en CVA na MICC met MiECC. Andere uitkomstmaten van deze studie zijn de levenskwaliteit van de patiënt en de patiënttevredenheid. Het studie design is opgesteld volgens de richtlijnen van de ‘1995 statement of consensus on assessment of neurobehavioral outcomes after cardiac surgery’.

1. **Doel van de studie**

Het in kaart brengen van de incidentie van delier, POCD en CVA na MICC. Aan de hand hiervan kan er zich een beter begrip vormen van hoe MICC invloed heeft op de postoperatieve neurologische outcome. Verder willen we graag de levenskwaliteit van de patiënt en de patiënttevredenheid na MICC in kaart brengen evenals de incidentie van angst en depressie na MICC.

1. **Eindpunten**
   1. **Primaire eindpunten**

De incidentie van POCD, delier en CVA na minimaal invasieve cardiale chirurgie.

- 1. **Secundaire eindpunten**
- De levenskwaliteit (quality of life) van de patiënt.
- De tevredenheid van de patiënt ten opzichte van de operatie en de uitgevoerde testen.
- De invloed van een aantal demografische en medische variabelen op de neurologische uitkomst na MICC.
- Incidentie van angst en depressie na MICC

# Studie opzet

Het betreft een monocentrische, prospectieve cohort studie. Er zal gebruikt gemaakt worden van 3 groepen: een Endo-CABG groep, een vergelijkingsgroep (PCI-groep) en een gezonde controle groep.

De gezonde controlegroep wordt geïncludeerd om de natuurlijke variatie die waargenomen wordt bij herhaald afnemen van neurofysiologische testen evenals een eventueel leereffect te kunnen elimineren.

De vergelijkingsgroep met patiënten die een percutane coronaire interventie (PCI) hebben ondergaan, wordt geïncludeerd om na te gaan of de waargenomen incidentie van neurologische complicaties niet kan toegeschreven worden aan de onderliggende cardiale voorgeschiedenis of aan de cardiale procedure die de patiënt ondergaat.

# Studiepopulatie

# Populatie en power analyse

Er worden drie groepen geïncludeerd: 1 groep bestaat uit patiënten die een endo-CABG ondergaan. Een 2^e^ groep bestaat uit patiënten die een PCI ondergaan. Een 3^e^ groep bestaat uit gezonde vrijwilligers.

De populatiegrootte werd berekenend aan de hand van een power-analyse op basis van het artikel van Ottens et al. (24). Er werd gebruik gemaakt van een significantie niveau van 5%, waarbij we een power van 90% verwachten met een standaardafwijking van 3.567. Het totaal aantal benodigde patiënten komt dan uit op 132. Dit betekent dat we voor elke groep 44 patiënten zullen nodig hebben. Rekening houdend met een drop-out tot 27% in elke groep, worden er 60 patiënten per studiegroep geïncludeerd.

Bij de verschillende groepen zal gebruik gemaakt worden van specifieke inclusie- en exclusie- criteria om een homogene patiëntenpopulatie te bekomen. Verder zal er ook demografische data van de deelnemers verzameld worden.

- 1. **inclusiecriteria**
- Een leeftijd van minstens 18 jaar
- Electieve Endo-CABG ingreep (groep1)
- Electieve PCI ingreep (groep 2)
- Gezonde vrijwilliger (groep 3)
  1. **exclusiecrieria**
- Medische voorgeschiedenis van:
  - beroerte of POCD
  - Symptomatisch carotislijden
  - Dementie
  - Renale dysfunctie 🡪 Glomerulaire filtratiesnelheid (GFR) < 30 ml/min
  - Hepatische dysfunctie 🡪 Serum glutamisch-oxaloacetisch transaminase (SGOT)/aspartaat aminotransferase (AST), of serum glutamisch-pyruvisch transaminase (SGPT)/alanine aminotransferase (ALT), meer dan drie keer boven de normale limieten
- Voorgeschiedenis van drugs-, medicatie- of alcoholmisbruik
- Aanwezigheid van een taalbarrière, onmogelijkheid tot communiceren met het subject
- Fysieke afwijking die de deelname aan de testen of het afwerken ervan niet toelaat
- Andere factoren die aanwijzen dat een successvolle neurocognitieve meting onmogelijk is
- een chirurgische revisie of intra-operatief majeur cardiaal event (Endo-CABG)
- Conversie naar cardiale chirurgie of een majeur cardiaal event (PCI)
- Simultane klepchirurgie (alleen voor MICC)
- Onmogelijkheid om zich te verplaatsen voor het follow-up contact
  1. **Demografische gegevens**

De volgende demografische gegevens zullen verzameld worden:

- leeftijd
- geslacht
- educatieniveau
- lengte, gewicht en BMI
- ASA classificatie

# Studietesten

De testen die uitgevoerd zullen worden zijn van een niet-invasieve aard en zullen gebruikt worden om neurocognitieve data van de patiënten de verzamelen. Een batterij van neurocognitieve testen zal gebruikt worden conform ‘the statement of consensus on assessment of neurobehavioural outcomes after cardiac surgery (1995)’om de POCD status te testen Er zal telkens van elke POCD test een baseline waarde bepaald worden pre-operatief en een waarde post-operatief.

Er zal een baseline neurologische onderzoek uitgevoerd worden bij elke patiënt door een neuroloog, evenals een postoperatief neurologisch onderzoek. Bij klinische aanwijzingen voor een CVA zal een CT-scan uitgevoerd worden om een CVA te objectiveren.

- 1. **Pre-operatief**
- Baseline neurocognitieve status: Mini Mental Test
- Baseline EQ-5D: quality of life
- Baseline angst en depressie
- Pre-operatief neurologisch klinisch onderzoek
- Baseline POCD status:
  - Rey auditory verbal learning test voor het verbale geheugen
  - Trailmaking A + B voor aandacht, concentratie en cognitieve verwerkingssnelheid
  - Grooved pegboard test voor fijne motor functies
  - Digit symbol-coding test voor verwerkingssnelheid en geheugen
  - Digit span test voor korte termijn verbaal geheugen

Alle baselinetesten zullen minimaal twee dagen voor de chirurgische dan wel de cardiale procedure worden afgenomen om een eventuele invloed van angst voor de procedure op de neurologische testen te minimaliseren.

- 1. **Post-operatief**
- EQ-5D: quality of life ((3 maanden post-operatief)
- Post-operatief neurlogisch klinisch onderzoek 🡪 CT-scan bij inidcatie van CVA (voor ontslag van intensieve zorgen)
- angst en depressie (3 maanden post-operatief)
- POCD status follow-up: (3 maanden post-operatief)
  - Rey auditory verbal learning test voor het verbale geheugen
  - Trailmaking A + B voor aandacht, concentratie en cognitieve verwerkingssnelheid
  - Grooved pegboard test voor fijne motor functies
  - Digit symbol-coding test voor verwerkingssnelheid en geheugen
  - Digit span test voor korte termijn verbaal geheugen

Postoperatief delier wordt reeds standaard geobjectiveerd bij opname op dienst intensieve zorgen na MICC door middel van de “Confusion Assessment Method” (CAM). De incidentie van delier zal als primair eindpunt worden meegenomen in deze studie.

# Studie analyse

- 1. **Definities en Analytische methodes**

POCD wordt in deze studie gedefinieerd als een afname in score tussen baseline en follow-up neurofysiologische testen die groter is dan de natuurlijke variatie waarbij tevens rekening wordt gehouden met een mogelijk leereffect. Hiervoor zal gebruik gemaakt worden van de zogenaamde Z-score (of Reliable Change Index (RCI)). Een Z-score is een dimensieloze eenheid die aangeeft hoe fors een verschil in score tussen baseline en vervolg test van 1 individu afwijkt (uitgedrukt in standaard deviatie) van de gemiddelde prestatie van een controlegroep (Gwylim Loyd).

In concreto zal de RCI van elke individuele patiënt berekend worden door achtereenvolgens 2 stappen uit te voeren:

1. Ten eerste zal voor elke patiënt de verschilscore van elke test berekend worden door de vervolg score af te trekken van de baseline score (Δx). Van deze Δx wordt vervolgens de gemiddelde verschilscore van deze test uit de controlegroep (=Δxc) afgetrokken om een eventueel leereffect uit te sluiten.
2. Vervolgens zal deze uitkomst gedeeld worden door de SD van Δxc om te corrigeren voor het effect van de natuurlijke variatie (de zogenaamde Z-score).

De RCI is het resultaat van de som van de Z-scores van alle testen.

POCD in een individuele patiënt wordt gedefinieerd als een een RCI kleiner of gelijk aan -1.96 (significantieniveau 5%) of 2 Z-scores van individuele testen kleiner of gelijk aan -1.96. Patiënten met een bewezen CVA worden automatisch geclassificeerd in de POCD groep.

Een CVA wordt gedefinieerd als een acuut neurologisch event waarbij er op CT of MRI hersenen tekens zijn van recente infarctzones.

Delier wordt gedefinieerd aan de hand van de CAM-ICU en het resultaat wordt gedichotomiseerd (wel of niet).

De groepen zullen onderling vergeleken worden via ANOVA testen of Kruskal-wallis testen, welke meer toepasselijk blijkt. De quality of life zal getest worden aan de hand van de Mann-whitney U test met significantie niveau 5%.

# Peri-operatieve procedure

In een poging om de incidentie van neurologische complicaties na MICC te reduceren, is in 2016 in het JESSA ziekenhuis Hasselt een protocol opgestart bestaande uit een reeks interventies waarvan bewezen is dat deze het risico op neurologische complicaties verlagen. Tevens heeft men de peri-operatieve procedure gestandaardiseerd om een zo homogene studiepopulatie te bekomen. Hieronder vindt u een overzicht van de gevolgde procedure. Bemerk dat we in het JESSA ziekenhuis gebruik maken van een minimaal invasief extra-corporeel systeem (Minimal invasive Extra-Corporeal Circulation of MiECC).

# 8.1 Intra-operative management

## Anesthesia:

All patients will receive premedication (alprazolam 0.25-0.5mg) one hour before arrival in the operating theatre. Induction of anesthesia will be performed with intravenous sufentanil (0.2-0.3 μg/kg) and propofol (1-2 mg/kg). Muscle relaxation will be achieved with cisatracurium (0.15-0.2mg/kg). After induction, anesthesia will be maintained with a continuous infusion of propofol (2-4mg/kg/h) and remifentanil (0.15-0.25μg/kg/’) and supplements of sufentanil (1-2microgr/kg) and cisatracurium as required. During cardiopulmonary bypass (CPB), sevoflurane inhalation (0,5-1%) will be used to maintain mean arterial pressure < 80-90 mmHg. Endotracheal intubation will be achieved with a bronchial blocker (EZ-Blocker, Teleflex) or a double lumen endotracheal tube (Mallinckrodt DLT) for single, separate lung ventilation during thoracoscopic harvesting of the internal mammary arteries.

Patients will be routinely monitored with a central venous and arterial line, continuous cardiac output monitoring by pulmonary artery catheter (Vigilance, Edwards Lifesciences) or in a semi-invasive way (FloTrack/Vigileo,Edwards Lifesciences) and cerebral oxygen saturation with near-infrared spectroscopy (Niro 200-SX, Hamamatsu) from pre-induction until completion of surgery. A full dose of heparin (300 IU/kg) will be administered in both groups and activated clotting time (ACT) will be maintained > 400 seconds. Tranexamic acid will be used as an antifibrinolytic agent. At the end of the procedure heparin will be reversed with protamine at a 1:1 equivalent dosage. Use of inotropic support (dobutamine), vasopression (norepinephrine) and vasodilation (milrinone) will be guided using hemodynamic data and TEE. Thorough transesophageal echocardiography (TEE) examination proves to be crucial in the management of patients with multivessel disease undergoing Minimal Invasive Cardiac Surgery (MICS) with Minimal invasive Extra-Corporeal Circulation (MiECC) as for preoperative evaluation, cannulation and perioperative management. Patients with severe atherosclerotic disease grade IV or V in the arch or ascending aorta on intraoperative TEE will be excluded from retrograde aortic perfusion and will be switched to central cannulation with antegrade perfusion (right subclavian artery) due to the risk of stroke.

## Surgical procedure:

Insufflation with carbon dioxide (controlled pneumothorax) will be used to ensure adequate visualization of the structures and to provide sufficient space for harvesting both internal mammary arteries during single lung ventilation. Full arterial revascularization will be performed through an anterolateral thoracotomy (4-5 cm incision). In multi vessel coronary artery disease, a Y-graft will be created by using the right mammary artery as free graft onto the left mammary artery.

Cardioplegia will consist of single shot antegrade cold (4°C) mixed cardioplegia 3:1 (blood: crystalloid, Fresenius Kabi, Schelle, Belgium) and will be administered through the aortic root vent. The composition of the crystalloid cardioplegia solution is shown in Table 1. Rectal temperature will be strictly maintained at 35.5-36.5°C and blood glucose levels will be controlled with an infusion of short-acting insulin. Warm blood (‘hot shot’) will be administered via the aortic root before removing the transthoracic clamp (Chitwood).

Table 1: Crystalloid cardioplegic solution

| Blood:Crystalloid cardioplegia (3:1) |
| --- |
| Sodium 40 mmol/L  Magnesium 76 mmol/L  Chloride 262 mmol/L  Potassium 62 mmol/L  Calcium 1 mmol/L  Procaine 5 mmol/L  Aqua ad 500 mL |

## Cardiopulmonary bypass

CPB in the MiECC group consists of a totally closed phosphorylcholine coated circuit with Revolution centrifugal pump (Sorin S.p.A., Mirandola, Italy), Inspire microporous hollow fibre membrane oxygenator (Sorin S.p.A., Mirandola, Italy) with integrated arterial filter and venous bubble trap (Sorin S.p.A., Mirandola, Italy). The system will be primed with 1000 ml of Plasma-Lyte® A (Baxter International Inc., Deerfield, IL, USA) without heparin. The priming solution will be evacuated out of the circuit and replaced by the patient’s blood to minimize haemodilution. Due to this intervention, no heparin will be added to the priming solution. De-priming of arterial and venous lines reduces priming volume to 300 ml. A blood collection bag will be integrated in the circuit, should positioning of the patient be insufficient in coping with excessive volumes. No open reservoir will be present. Cell saver drainage will be used for intra-pericardial bloodshed. Aortic root venting will run via the upper bubble trap to minimize back-bleeding in coronary arteries. Non-pulsatile flow at 1.8-2.4 L/min/m2 will be maintained with mean arterial pressure between 70-80 mmHg with norepinephrine or sevoflurane. PH will be managed according to the alpha-stat principle.

# 8.2 Postoperative management:

All patients will be transferred to the intensive care unit (ICU) and will be extubated depending on clinical criteria within 2-6 h after surgery. For postoperative analgesia, we will use a continuous infusion of local anesthetics (ropivacaine 0.2%,6 ml/h) through a catheter in the intercostal and paravertebral space for 3 days. The catheter will be placed by the surgeon under endoscopic guidance at the end of the procedure and a bolus (ropivacaine 0.5 %,10 ml) will be administered.

Postoperative analgesia will be further provided with intravenous paracetamol and a continuous infusion of piritramid. Hyperthermia will be strictly avoided and blood glucose levels will be controlled with an infusion of short-acting insulin.

On our ICU, a program for prevention of postoperative delirium was implemented, consisting of minimal sedation, avoidance of benzodiazepines and early mobilization. Our nursing staff succeeded in creating a healing environment in a patient-centered approach with interventions for re-orientation, improvement of sensory input (visual and hearing aids) and non-pharmacological sleep enhancement (noise and light reduction, less nursing interventions at night).

# Ethische overwegingen en wettelijk kader

Deze studie zal voldoen aan sectie 10, subsectie 1 van de wet Wetenschappelijk Medisch Onderzoek(WMO): “the investigator will inform the subjects and the reviewing accredited METC if anything occurs, on the basis of which it appears that the disadvantages of participation may be significantly greater than was foreseen in the research proposal. The study will be suspended pending further review by the accredited METC, except insofar as suspension would jeopardise the subjects’ health. The investigator will take care that all subjects are kept informed.”

Verder respecteert deze studie de ethische principes zoals beschreven in de Verklaring van Helsinki:

“WORLD MEDICAL ASSOCIATION DECLARATION OF HELSINKI”

Ethical Principles for Medical Research Involving Human Subjects

Adopted by the 18th WMA General Assembly, Helsinki, Finland, June 1964, and amended by the

29th WMA General Assembly, Tokyo, Japan, October 1975

35th WMA General Assembly, Venice, Italy, October 1983

41st WMA General Assembly, Hong Kong, September 1989

48th WMA General Assembly, Somerset West, Republic of South Africa, October 1996 and the 52nd WMA General Assembly, Edinburgh, Scotland, October 2000

Note of Clarification on Paragraph 29 added by the WMA General Assembly, Washington 2002

Note of Clarification on Paragraph 30 added by the WMA General Assembly, Tokyo 2004”

1. **Bijlagen**

## Vragenlijst angst
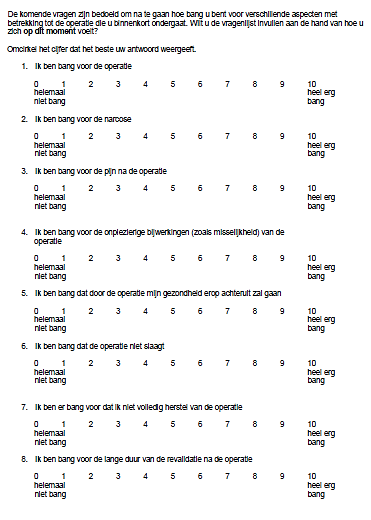


**Vragenlijst depressie**


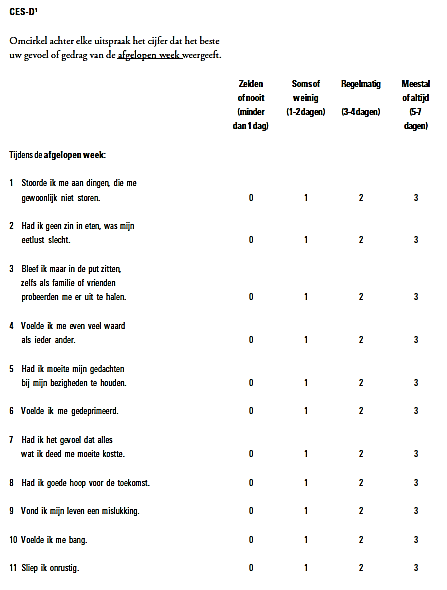


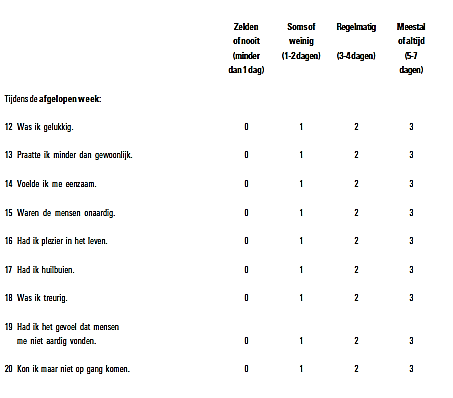


1. **Referenties**
2. likosky DS NW, Ross CS. Improving outcomes of cardiac surgery through cooperative efforts: the northern new England experience. Seminars in Cardiothoracic and Vascular anesthesia. 2005;9:119-21.
3. S. G. Cerebral disorders after open heart operations. New England Journal of medicine. 1965;272:489-98.
4. Furlan AJ, Sila CA, Chimowitz MI, Jones SC. Neurologic complications related to cardiac surgery. Neurol Clin. 1992;10(1):145-66.
5. Stamou SC. Stroke and encephalopathy after cardiac surgery: the search for the holy grail. Stroke. 2006;37(2):284-5.
6. Jensen BO HP, Rasmussen LS, Pedersen PU,Steinbruchel DA. health-related quality of life following off-pump versus on-pump coronary artery bypass grafting in elderly moderate to high-risk patients: a randomized trial European Journal Cardio-thoracic Surgery. 2006;30:294-9.
7. Newman MF, Grocott HP, Mathew JP, White WD, Landolfo K, Reves JG, et al. Report of the substudy assessing the impact of neurocognitive function on quality of life 5 years after cardiac surgery. Stroke. 2001;32(12):2874-81.
8. Rumsfeld JS, Magid DJ, O'Brien M, McCarthy M, Jr., MaWhinney S, Scd, et al. Changes in health-related quality of life following coronary artery bypass graft surgery. Ann Thorac Surg. 2001;72(6):2026-32.
9. Newman MF. Open heart surgery and cognitive decline. Cleve Clin J Med. 2007;74 Suppl 1:S52-5.
10. Uysal S, Reich DL. Neurocognitive outcomes of cardiac surgery. J Cardiothorac Vasc Anesth. 2013;27(5):958-71.
11. Dafydd GL DM, Marcela PV. cognitive decline after anaesthesia and critical care. British journal of anaesthesia. 2012.
12. Ottens TH, Dieleman JM, Sauer AM, Peelen LM, Nierich AP, de Groot WJ, et al. Effects of dexamethasone on cognitive decline after cardiac surgery: a randomized clinical trial. Anesthesiology. 2014;121(3):492-500.
13. Stygall J, Newman SP, Fitzgerald G, Steed L, Mulligan K, Arrowsmith JE, et al. Cognitive change 5 years after coronary artery bypass surgery. Health Psychol. 2003;22(6):579-86.
14. Ganushchak YM, Fransen EJ, Visser C, De Jong DS, Maessen JG. Neurological complications after coronary artery bypass grafting related to the performance of cardiopulmonary bypass. Chest. 2004;125(6):2196-205.
15. Newman MF, Mathew JP, Grocott HP, Mackensen GB, Monk T, Welsh-Bohmer KA, et al. Central nervous system injury associated with cardiac surgery. Lancet. 2006;368(9536):694-703.
16. Selnes OA, Gottesman RF, Grega MA, Baumgartner WA, Zeger SL, McKhann GM. Cognitive and neurologic outcomes after coronary-artery bypass surgery. N Engl J Med. 2012;366(3):250-7.
17. Nadelson MR, Sanders RD, Avidan MS. Perioperative cognitive trajectory in adults. Br J Anaesth. 2014;112(3):440-51.
18. Steinmetz J, Christensen KB, Lund T, Lohse N, Rasmussen LS, Group I. Long-term consequences of postoperative cognitive dysfunction. Anesthesiology. 2009;110(3):548-55.
19. Slater JP, Guarino T, Stack J, Vinod K, Bustami RT, Brown JM, 3rd, et al. Cerebral oxygen desaturation predicts cognitive decline and longer hospital stay after cardiac surgery. Ann Thorac Surg. 2009;87(1):36-44; discussion -5.
20. Modi P, Chitwood WR, Jr. Retrograde femoral arterial perfusion and stroke risk during minimally invasive mitral valve surgery: is there cause for concern? Ann Cardiothorac Surg. 2013;2(6):E1.
21. John M. Murkin SPN, David A. Stump, James A. Blumenthal. statement of concensus on assessment of neurobehavioral outcomes after cardiac surgery. Ann Thorac Surg. 1995;59:1289-95.
22. Knights RM, Moule AD. Normative data on the motor steadiness battery for children. Percept Mot Skills. 1968;26(2):643-50.
23. Powell JB, Cripe LI, Dodrill CB. Assessment of brain impairment with the Rey Auditory Verbal Learning Test: a comparison with other neuropsychological measures. Arch Clin Neuropsychol. 1991;6(4):241-9.
24. Reitan RM. The relation of the trail making test to organic brain damage. J Consult Psychol. 1955;19(5):393-4.
25. Ottens TH, Dieleman JM, Sauer AM, Peelen LM, Nierich AP, de Groot WJ, et al. Effects of dexamethasone on cognitive decline after cardiac surgery: a randomized clinical trial. Anesthesiology. 2014;121(3):492-500.
